# Community Inclusion in PrEP Demonstration Projects: Lessons for Scaling Up

**DOI:** 10.12688/gatesopenres.13042.2

**Published:** 2019-12-17

**Authors:** Sushena Reza-Paul, Lisa Lazarus, Smarajit Jana, Protim Ray, Nelly Mugo, Kenneth Ngure, Morenike Oluwatoyin Folayan, Florita Durueke, John Idoko, Luc Béhanzin, Michel Alary, Daouda Gueye, Moussa Sarr, Wanjiru Mukoma, Jordan K. Kyongo, Rutendo Bothma, Robyn Eakle, Gina Dallabetta, Josie Presley, Robert Lorway

**Affiliations:** 1 Centre for Global Public Health, Rady Faculty of Health Sciences, Community Health Sciences, University of Manitoba, Winnipeg, Canada; 2 Ashodaya Samithi, Mysore, India; 3 Durbar Mahila Samanawaya Committee, Kolkata, India; 4 Center for Clinical Research, Kenya Medical Research Institute, Nairobi, Kenya; 5 Department of Global Health, University of Washington, Seattle, USA; 6 Department of Community Health, Jomo Kenyatta University of Agriculture and Technology, Nairobi, Kenya; 7 Department of Child Dental Health, Obafemi Awolowo University, Ile-Ife, Nigeria; 8 New HIV Vaccine and Microbicide Advocacy Society, Lagos, Nigeria; 9 Department of Medicine, University of Jos, Jos, Nigeria; 10 Centre de recherche du CHU de Québec, Université Laval, Quebec, Canada; 11 Dispensaire IST, Centre de santé communal de Cotonou 1, Cotonou, Benin; 12 École Nationale de Formation des Techniciens Supérieurs en Santé Publique et en Surveillance Épidémiologique, Université de Parakou, Parakou, Benin; 13 Département de médecine sociale et préventive, Université Laval, Quebec, Canada; 14 Institut national de santé publique du Québec, Quebec, Canada; 15 Institut de Recherche en Santé, de Surveillance Epidémiologique et de Formation (IRESSEF), Dakar, Senegal; 16 Westat, Inc, Rockville, USA; 17 LVCT Health, Nairobi, Kenya; 18 Wits Reproductive Health and HIV Institute (WRHI), Johannesburg, South Africa; 19 Faculty of Public Health and Policy, London School of Hygiene and Tropical Medicine, London, UK; 20 Bill & Melinda Gates Foundation, Washington, USA; 21 Bill & Melinda Gates Foundation, Seattle, USA

**Keywords:** pre-exposure prophylaxis, demonstration projects, HIV, prevention, sex work, serodiscordant couples

## Abstract

Pre-exposure prophylaxis (PrEP) has emerged as a new HIV prevention strategy. A series of demonstration projects were conducted to explore the use of PrEP outside of clinical trial settings. Learning from the failures in community consultation and involvement in early oral tenofovir trials, these PrEP projects worked to better engage communities and create spaces for community involvement in the planning and roll out of these projects. We describe the community engagement strategies employed by seven Bill & Melinda Gates Foundation-funded PrEP demonstration projects. Community engagement has emerged as a critical factor for education, demand generation, dispelling rumors, and supporting adherence and follow up in the PrEP demonstration project case studies. The increasing global interest in PrEP necessitates understanding how to conduct community engagement for PrEP implementation in different settings as part of combination HIV prevention.

## Background

Pre-exposure prophylaxis (PrEP) is a new HIV prevention intervention for people who consider themselves at risk of acquiring HIV ^1, 2^. Current protocols and guidelines outline PrEP as the use of a daily pill containing the combination of the antiretroviral therapies tenofovir and emtricitabine (the original formula for tenofovir known as TDF) ^1^. In September 2015, the World Health Organization (WHO) recommended that people at “substantial risk” of HIV infection have access to PrEP as part of comprehensive HIV prevention strategies ^3^.

A number of randomized control trials have demonstrated clinical efficacy of PrEP ^4– 11^. However, early oral tenofovir trials among marginalized communities, including in Cambodia, Cameroon, Nigeria, and Malawi among female sex workers (FSWs) and in Thailand among people who inject drugs, drew international attention with concerns over ethical violations in research practices ^12– 14^. Organized community action led to a halt of the trials in Cambodia and Cameroon due to concerns about the standard of prevention and care ^12^. Trials failed to start in Malawi because of ethical concerns about access to care for study participants who HIV seroconverted and developed resistance to HIV treatment due to their PrEP use ^15^. Although significant concerns were raised about the Thai trial, it ultimately proceeded ^16^, while the trial in Nigeria was discontinued by the research team based on non-protocol adherence ^17^.

The community actions articulated that each of these trials was unethically conducted on the basis of insufficient collaboration with communities, providing selective information about potential adverse effects, and being unwilling to provide comprehensive health insurance and care for participants ^12– 14^. In order to find ways to move towards a more collaborative approach to biomedical HIV prevention research, a series of consultations following these study closures led to the creation of the *Good Participatory Practice: Guidelines for biomedical HIV prevention trials* ^18^. These guidelines, prepared by UNAIDS and AIDS Vaccine Advocacy Coalition (AVAC), highlight community engagement in biomedical HIV prevention clinical trials as an ethical imperative. This was further stressed in an additional guidance note prepared by UNAIDS and the World Health Organization on *Ethical considerations in biomedical HIV prevention trials* ^19^.

These guidelines and the lessons learned from the initial controversies paved the way for more inclusive research practices, also bridging research into implementation practice. PrEP clinical trials and demonstration projects designed and implemented after these controversies were more proactive in their community engagement practices, engaging communities and creating spaces for community participation from planning through implementation of these projects. With confirmation of PrEP efficacy, the WHO called for implementation studies to inform product effectiveness and implementation. The Bill and Melinda Gates Foundation (BMGF) funded several demonstration projects with the aim of providing data on the implementation of PrEP in a diverse range of settings and populations. The aim of this paper is to articulate the community engagement practices implemented in seven BMGF-funded demonstration projects (listed in Table 1). We describe the use of various community engagement strategies and engagement with a wide range of stakeholders in a diverse range of settings and populations in order to help inform community engagement in ongoing PrEP scale up globally.

**Table 1.** List of PrEP Demonstration Projects funded by the Bill and Melinda Gates Foundation.

Study Name	City, Country	Population	Implementer
The Partners Demonstration Project: Kenya and Uganda	Kenya: Thika & Kisumu, Uganda: Kampala & Kabwohe	HIV-1 serodiscordant couples	Kenya Medical Research Institute (KEMRI),Nairobi, Kenya Partners in Health Research and Development (KEMRI),Thika Infectious Diseases Institute (IDI), Makerere University, Kampala, Uganda Clinical Research Center, Kabwohe, Uganda
A demonstration project of antiretroviral-based HIV prevention among HIV-1 sero-discordant couples in Nigeria	Nigeria	HIV-1 serodiscordant couples	The New HIV Vaccine and Microbicide Advocacy Society (NHVMAS) on behalf of the National Agency for the Control of AIDS
Early antiretroviral therapy and pre-exposure prophylaxis for HIV prevention among female sex workers in Benin, West Africa	Cotonou city, Benin	Female sex workers	CHU de Québec – Université Laval
A demonstration HIV pre-exposure prophylaxis (PrEP) project with FTC/TDF among female sex workers in Dakar, Senegal	Dakar, Senegal	Female sex workers	IRESSEF/WESTAT
The IPCP (Introducing PrEP into HIV Combination Prevention) Project: Kenya	Nairobi, Kisumu and Homabay, Kenya	Female sex workers, men who have sex with men, young women	LVCT Health Bar Hostess Empowerment and Support Programme (BHESP) * Health Options for Young Men on HIV/AIDS/STI (HOYMAS) *
The treatment and prevention for female sex workers (TAPS) demonstration project: South Africa	Johannesburg and Pretoria, South Africa	Female sex workers	WITS Reproductive Health and HIV Institute (Wits RHI)
Closing a critical HIV prevention gap: demonstrating safety and effective delivery of daily oral pre-exposure prophylaxis (PrEP) as part of an HIV combination preventive intervention for sex workers in Kolkata and Mysore-Mandya, India	Kolkata and Mysore-Mandya, India	Female sex workers	University of Manitoba *Durbar Mahila Samanwaya* Committee (DMSC) * Ashodaya Samithi *

* Denotes community-based organization

## Assessment of the research projects

Throughout the course of the demonstration projects, BMGF conducted meetings involving implementers from the different project sites. The purpose of these meetings was to share project updates and facilitate cross-learning. The possibility of joint publications were discussed in these meetings and crystalized during a multi-site meeting in Benin in 2016. During this meeting, a joint manuscript was planned to capture the community engagement strategies employed in the various demonstration project sites.

Members of each of the seven research projects completed an open-ended questionnaire exploring the objectives of their project, demand generation strategies, and role of the community in planning, recruitment, follow up, and retention. The first and second authors then conducted follow up phone interviews with a number of the sites to clarify their responses and gather more details about their work. A draft case study of each site was prepared, shared with the partners, and finalized with their inputs.

## Findings: The role of community in PrEP demonstration projects

We present mini-case studies of each project that detail the overall role of community in various stages of the projects, challenges, and achievements related to community involvement. While we recognize that community can mean different things in different contexts, for the purpose of the case studies presented, community includes the populations targeted by the demonstration projects (serodiscordant couples, sex workers, men who have sex with men, and young women) and the key stakeholders working with these populations.

## Demonstration projects involving serodiscordant couples

### The Partners Demonstration Project: Kenya and Uganda

The Partners Demonstration Project explored delivery approaches among HIV-1 serodiscordant couples in Kenya and Uganda, with two sites in each country. PrEP was offered to the HIV-uninfected partner until the partner living with HIV had initiated and used ART for at least 6 months. The project ran from November 2012 to June 2016 and enrolled 1013 HIV serodiscordant couples across sites ^20– 22^.

Community engagement was conceptualized through previous experience with the community during the Partners PrEP trial ^4^. The project’s community strategy involved engagement with a wide range of stakeholders, community education on PrEP, and dispelling myths about HIV serodiscordance. Approval for the project was initially sought from national and local government authorities in the Ministry of Health and Public Works, which was followed by meetings with lead members of community organizations (non-governmental, community-based, and faith-based), HIV serodiscordant couples support groups, and national and local health care authorities. The project sites instituted community advisory groups (CAGs), comprised of representatives of the above groups and members representing HIV serodiscordant couples. The members were selected through several steps, which included stakeholder analysis to identify the key people who reflected the potential interests of the project participants. After developing a complete listing of stakeholders, the team identified those representatives key to the HIV prevention agenda and created a rapport with them before moving to invite them to join the CAG. This engagement process was guided by site-specific standard operating procedures for the development of CAGs. The CAGs were formed to include 12 members and acted as a link between the community and the project. They provided input on the study implementation, reviewed and advised the proposed recruitment approaches, and addressed community concerns. The research team worked with stakeholders to dispel myths, rumors, social taboos, concerns, and misconceptions related to HIV serodiscordance.

Community health workers (CHWs) were the backbone to community entry. CHWs already exist under the community health strategy, where each CHW provides a range of health services to 20 households ^23^. The CHWs are well respected in the community and have worked on previous studies, including the Partners PrEP trial, thus had already worked to build trust with the community over many years. The project team worked with all the available CHWs in the communities where recruitment occurred. CHWs conducted outreach and built trust about the project among the community. The project teams grouped CHWs by region and held training sessions on PrEP and HIV serodiscordance. During project implementation, the CHWs discussed challenges faced by HIV serodiscordant couples and proposed strategies for recruitment. The HIV serodiscordant couple groups and other civil society organizations were agents who interacted with the local communities creating a receptive environment. Various leaders (opinion, religious, and others) supported couples HIV testing. Radio and television stations were used to reach a wider audience with the benefits of couple testing and PrEP as a prevention option. There were regular engagements with various stakeholders on the safety and efficacy of PrEP.

Recruitment strategies utilized a multi-prong approach that included capacity building of the CHWs, community education through posters and flyers that emphasized the benefits of couples HIV counseling and testing (CHCT), and community mobilization by peer recruiters and CHWs through the use of skits, music, puppets, and dances. Special attention was taken to develop messages for men who were generally more reluctant to go for testing. Recruitment strategies included referrals from existing voluntary counseling and testing centers, public promotion of CHCT by well-known individuals, community organizations (such as churches), and mobilization around events (for example, around Valentine’s Day and market days). Discordant couples support groups used to meet regularly to promote further participation, retention, discuss challenges, and means of addressing them, including rumors.

Challenges around recruitment included: (1) low HIV testing at voluntary counseling and testing centers lacking a continuous supply of HIV testing kits and (2) a shortage of counselors with skills to counsel and test couples for HIV. This led to a low number of couples being tested and therefore few discordant couples being identified. Increases in clinic efficiency would likely lead to higher retention among couples.

The Partners Demonstration Project was able to recruit within the timelines of the project and achieved high retention rates and demonstrated higher effectiveness of PrEP than what was observed in the Partners PrEP trial. This evidence from the Demonstration Project was key to the rollout of PrEP by the Ministry of Health. The Kenya Ministry of Health has since rolled out PrEP for persons at risk of HIV, including HIV-serodiscordant couples, as part of its combination strategy ^24^. Currently, there are over 50,000 people who have initiated PrEP in Kenya. PrEP in Uganda continues to be made available through a number of ongoing projects ^25^.

## The Nigeria PrEP Demonstration Project

The Nigeria PrEP Demonstration Project aimed at providing PrEP to HIV-negative partners in HIV-1 serodiscordant relationships until the HIV-positive partner was virologically suppressed. It used community-designed models that recruited 345 HIV-1 serodiscordant couples across three main sites and six satellite sites in Nigeria. The study took place between 2012 and 2018 and was designed through stakeholder consultation that engaged HIV service providers, consumers, policymakers, as well as other key stakeholders in the design and implementation of the project. The project first had a roadmap developed through stakeholder engagement ^26^. The roadmap was divided into three phases. The first phase involved a modeling study to determine if PrEP would be of benefit to the national HIV prevention program ^27^. The second phase included formative research to help determine the study design and the study target population ^28^. The third phase rolled out the design and implementation of the main demonstration project.

The main objective of the demonstration project was to determine the effectiveness of PrEP delivery models in reducing HIV transmission among HIV-1 serodiscordant couples. The project also prioritized the formation of community engagement structures at the three sites. The primary role of the community engagement structure was to serve as a liaison between the research site and the community hosting the demonstration project. First, the New HIV Vaccine and Microbicide Advocacy Society (NHVMAS) developed and received ethical clearance for the implementation of a protocol for its community engagement project in line with the *Good Participatory Practice: Guidelines for biomedical HIV prevention trials* ^18^. Next, NHVMAS held a public engagement meeting with a wide range of stakeholders – policymakers, HIV program planners, community advocates, traditional leaders, religious leaders, journalists, and researchers. The stakeholders were introduced to the objectives and methodology of the PrEP demonstration project and the importance of a community engagement structure for the project was shared. NHVMAS, in collaboration with stakeholders present at the meeting, jointly designed the community engagement structure for each site. This included decisions on the community groups to be represented on the Community Advisory Board (CAB) and the civil societies to conduct the public education program. A community mapping exercise was done and decisions were taken on which area of the town each civil society organisation would cover to conduct public education about HIV prevention research in general, and the PrEP demonstration project specifically. The size of the CAB ranged from 15 to 19 members with members from community leaders, youth leaders, women leaders, religious leaders, journalists, representative of the State Agency for the control of AIDS, and representatives from the population of people living with HIV.

Letters were sent to the identified organizations and institutions to be represented on the CAB. Monthly meetings were facilitated by NHVMAS. The monthly meeting enabled the research sites to give updates to the CABs, CABs to give updates about their engagement with the community, and enabled NHVMAS to hold debrief meetings with the civil society organisations conducting public education programs. The boards had a standing operating procedure manual guiding their monthly meetings. The manual included details such as the expected agenda for the monthly meeting and clarifications about the roles and responsibility of CAB members and its leadership. Responsibilities of CAB members included the education of the constituencies they represent about the project, monthly constructive feedback to the research site about discussions and concerns raised by constituency members about the research, and feedback to the research site about the study implementation during the site monthly debriefs.

Representatives from the civil society were initially trained on the use of a developed and pilot-tested flipchart for educating the public about PrEP. They were also equipped with educational pouches that contained samples of biomedical HIV prevention tools discussed in the flipchart to show during public education programs. This included samples of PrEP. Monthly meetings were also held for the public educators to report back on targets reached during their public education programs and discuss questions asked and challenges faced during their public engagement. The issues were jointly resolved with NHVMAS. A major issue that arose were concerns about financial constraints to meet set targets for public education. This concern never disrupted the study process.

A quarterly research literacy capacity building training for CAB members and the civil society organization representatives was conducted at each of the research sites. A curriculum was developed for this educational program. Four two-day capacity building workshops were conducted at each of the study sites throughout the project, and included training on prevention technologies, research processes, and ethics. In addition, a social surveillance system was instituted by the project. Social surveillance was included in the study design to ensure semi-annual monitoring of individual, facility, and community concerns about the study. The surveillance was managed by two social scientists located at each of the study sites and coordinated by the two lead persons managed by the National Agency for the Control of AIDS (NACA). The results of their monitoring exercises were fed back in real time into the project implementation process. The one challenge raised by the social scientists was a concern related to poor counseling of the couples diagnosed as HIV sero-positive at one of the sites before enrolling them into the PrEP study. The social scientists felt that the couples where being too quickly enrolled into the study without time to process their diagnosis. The research team needed to work with the site team to discuss how to improve on the counseling of clients to support the news of an HIV-positive test result.

A media engagement project was conducted at one of the three sites. The aim was to assess the impact of public engagement through the use of the media on study participant recruitment and retention. Analysis showed that although the media engagement resulted in a significant increase in public inquiry at the study intervention site, it was not significantly associated with an increase in study participant recruitment or retention. The project led to an increase in awareness and public education about PrEP in Nigeria. Other community members, such as FSWs and MSM, took part in consultative meetings on how to maximize the study outcome to address PrEP needs. Stakeholders successfully mobilized and advocated for the inclusion of PrEP in national guidelines in 2016. Research findings were disseminated to community members at the annual civil society organization accountability forum in 2018.

## Demonstration projects involving sex workers

### Early antiretroviral therapy and PrEP for HIV prevention among FSWs: Benin, West Africa

The CHU de Québec – Université Laval Research Centre has been working with FSW projects in Benin since 1992. The PrEP demonstration project was aimed at assessing the feasibility and usefulness of integrating treatment as prevention (TasP) and PrEP to the combination prevention package offered to sex workers in Benin. The project was initiated from the end of 2014 until December 2016 in Cotonou. After a community preparedness phase, 256 FSWs were recruited for PrEP. All participants were followed up for 12 months.

Four community workers were mobilized and recruited collaboratively with and through the sex worker organization in Benin named Association Solidarité, which is Benin’s National AIDS Control Programme (NACP) partner. During the community preparedness phase, community workers (CWs) worked with FSWs and provided information on the project in small groups and in one-on-one sessions. CWs were field workers who had at least 15 years of experience in working with sex workers (but were not sex workers themselves). CWs carried out these activities in two teams. Each team was made up of one senior CW and one junior CW. The two senior CWs were leaders of an NGO that has been involved in HIV prevention research in the sex work milieu for the last two decades and the junior CWs were from an NGO recently involved in the sex work milieu for HIV prevention sensitization. Each team was responsible for covering one of the two zones forming the catchment area. This enabled the same CWs to closely follow up with participants and address their concerns throughout the study (screening, enrolment, and follow up). Before intervening in an area, CWs first contacted the owners or managers of the sex work sites (brothel, bar, street sex work, etc.) to determine the most suitable timings for their interventions. They then provided background information on HIV and about the project. Information about the study was also shared with Association Solidarité to garner support and facilitate mobilization to participate in the study. The mobilization for screening was carried out in groups of 7–10 followed by individual meetings. Simultaneously, peer educators (especially those working with project Équité en santé, another CHU de Québec – Université Laval-led HIV prevention project) also mobilized the FSWs who were part of their network.

During community mobilization, coupons for screening visits were handed out to the women. Active tracking was carried out for those who missed appointments. The CWs reminded the women of the appointment on three occasions, one week, three days, and one day prior to the appointment, either by telephone or by meeting them at their residence, workplace, or place of their choice. In case of a missed screening visit, participants were contacted to see if they were still willing to participate in the study and were provided with a new appointment. Often peer educators also reminded the women of their appointments by repeatedly visiting them. These peer educators worked as voluntary assistants to the CWs and provided advice and supported mobilization for the screening and recruitment visits. The work of the peer educators was not formalized in the project and they were therefore not compensated for their volunteered support.

Following the screening visit, an auxiliary nurse reviewed the recruitment and the CWs were responsible for contacting the participants to confirm the appointment. The CWs were also responsible for promoting adherence to PrEP through various educational community tools and informing the participants about side effects during their field visits for community-based distribution of condoms and PrEP, as well as locating women lost to follow up. The catchment area was divided into two zones, and this zoning proved important so that the community workers were present, visible, and accessible and could continuously give feedback and information to FSWs during their fieldwork. They were able to simultaneously addressed concerns in the community and find solutions to concerns or requests that FSWs presented. A main challenge during the project was the mobility of the FSWs, which was addressed through networking. Peer educators could also have been more formally integrated into the process.

Upon completion of the study, sex workers were involved in dissemination of the results. A dissemination workshop was conducted with various stakeholders including FSWs and policymakers. The success of the project was based on the broad participation of FSWs, health authorities, and the Vice squad who recognized the importance of avoiding police harassment of the FSWs and encouraged PrEP adherence. In the short-term following the workshop in May 2017, there was no government plan to scale up PrEP in Benin. However, it has been possible to provide PrEP to the study participants who still requested it and the Benin Ministry of Health is now in the process of implementing PrEP access for key populations and serodiscordant couples.

## The Senegal PrEP Demonstration Project

A PrEP demonstration project was conducted in Dakar, Senegal among FSWs. The project explored the feasibility of providing daily oral PrEP for 12 months to enrolled participants at four Ministry of Heath-run clinics. A total of 350 participants were screened during an accrual period of 3 months (from June 2015 to October 2015) and 267 eligible participants were enrolled.

The community has been involved in all phases of the project. The project team worked with the community to assess its interest in PrEP and identify barriers and facilitators to implementation. The team also worked with the community to foster their support in raising awareness about PrEP and adherence and retention issues.

The planning phase occurred over five weeks in 2015. The feasibility study provided information regarding sex workers’ perceptions on PrEP, its acceptability, and strategies to resolve challenges, including stigma. The feasibility study included activities such as focus group sessions with the FSW population and individual in-depth interview with key informants. The focus group sessions were organized with two groups: (1) registered FSWs and (2) unregistered FSWs. Individual in-depth interviews were organized with key informants who had more than five years experience working with FSWs and health personnel working in HIV control. The purpose of the interviews was to explore the perspectives and opinions of the study by policymakers, program managers, service and health care providers, and FSW community members and leaders. FSWs were approached for recruitment during their regular visits as registered sex workers in one of four selected health centers. Peer educators supported the recruitment of unregistered sex workers in their existing networks in neighborhoods or in sex work venues. Key informants were recruited with the support of NGO partners that have extensive experience working and or interacting with FSWs and health staff. Strategies to resolve barriers and challenges, such as stigma, were discussed.

Four peer educators per study site were recruited by the study team, in collaboration with local organizations working on HIV prevention and trained for the implementation phase. They were chosen based on their leadership within the sex worker community and their experience in HIV prevention efforts. Peer educators worked closely with the social workers at each of the study sites and facilitated recruitment and follow up. They also sensitized the health care staff on the needs of the community. They were tasked with identifying potential participants in the neighborhoods, provided them with information about PrEP, and referred interested participants to the nearest clinics where PrEP was provided. Social workers and midwives were responsible for informing sex workers about the study procedures. Peer educators played a key role designing communication strategies on PrEP. They were responsible for following up with participants in-person twice a week, along with phone calls, and home visits for those who missed appointments. During their interactions, they tried to find out the reasons for missed appointments, re-educated participants on PrEP, encouraged them to come in for routine clinic visits, and addressed issues related to stigma, family, and adherence. The peer educators who were taking PrEP became role models and shared their experiences with the participants. With time, participants were calling the free study phone number on their own to follow up on appointments.

Challenges arose during the project related to: (1) difficulty in recruiting unregistered FSWs as they do not regularly attend health centers; (2) the mobility of female sex workers who traveled and/or moved away from the study area; (3) community stigma related to daily pill taking and HIV, with FSWs taking PrEP thought to be living with HIV, which compromised their sex work negotiation capacity; (4) the heavy pill burden, (5) the long duration of study visits needed to perform all study activities; (5) stigma experienced at some health centers; and (6) challenges with blood sampling among FSW who might not be comfortable with blood. Challenges were addressed by adapting the patient-specific counseling approach by making recommendations that addressed the personal challenges faced by study participants to help them better adhere to the study procedures, Peer educators also played a major role in addressing challenges through close and regular follow up of FSWs. Similarly, discussion sessions were organized with FSWs and peer educators to better understand and address the challenges they faced, including stigmatization at all levels and family challenges.

Preliminary findings and scale up strategies have been shared at a national scientific meeting organized by the Ministry of Health. The Ministry of Health and Social Action, the National AIDS Control Program (NACP/CNLS), the Division for HIV/AIDS and STI Control, and NGOs for key population were all involved in dissemination events. Further dissemination is planned to advocate for the inclusion of PrEP as an HIV prevention option for the national program. PrEP national recommendations for key population are being developed. The availability of PrEP for populations such as FSWs and MSMs is being included in the upcoming National Strategic Plan. PrEP and related services are expected to soon be available in selected health facilities across the country.

## The Introducing PrEP into HIV Combination Prevention (IPCP) Project: Kenya

LVCT Health is an indigenous Kenyan non-governmental organization that provides HIV and sexual reproductive health services to FSWs, men who have sex with men (MSM), and young women (YW). This project aimed to demonstrate how daily oral PrEP can be delivered as part of an HIV combination prevention package among these three populations. Between August 2015 and October 2016, the project recruited 796 FSWs, 597 MSM, and 723 YW. Participants were offered daily oral PrEP and followed over a period of 12 months.

Community engagement was initiated in 2013 with a sub-study that explored the views on the feasibility of the introduction of PrEP in Kenya ^29^. Several community organizations assisted in the IPCP project, but two key populations organizations, Bar Hostess Empowerment and Support Programme (BHESP) and Health Options for Young Men on HIV/AIDS/STI (HOYMAS) were direct implementing partners. Two representatives of these two organizations were co-investigators on the project. Other community organizations included the Kenya Sex Workers Alliance (KESWA), Nyanza, Rift Valley and Western Kenya Network (NYARWEK) LGBTI Coalition, Ishtar MSM, and the Gay and Lesbian Coalition of Kenya (GALCK). These organizations were involved in various stages of the project. During the project design, the peer-led organizations were involved in: the selection of the implementation sites; providing leadership on community education on combination HIV prevention and PrEP, sharing community perspectives on PrEP with the project team, ensuring community buy-in, mobilizing community for participation, advising on strategies to address emerging community concerns regarding PrEP, and the constitution of a national-level CAB with members of various MSM and FSW organizations. The twelve CAB members were nominated by the community organizations that partnered with LVCT to implement the project.

During implementation, the CAB supported different aspects of the project, such as project monitoring, PrEP advocacy at the community and national level, and results dissemination and discussion. At site level, population-specific community advisory committees (CACs) were formed consisting of nominees from different MSM and FSW organizations, as well as representatives of YW from community-based organizations. Each CAC had representation in the national-level CAB and membership varied from 10-12 people per site. They held monthly meetings to develop and review demand generation strategies for PrEP, discuss adherence issues, develop materials on HIV prevention tailored to meet target group needs and strategies for addressing emerging community and user concerns. They acted as PrEP advocates to the communities they represented and also provided community feedback to the site in-charges where they were attached.

Peer educators were selected from existing HIV prevention programs (MSM and FSW) and from established community health volunteers of the same age group (18-29 years) for the YW. They were then trained on PrEP and used brochures and flyers to disseminate information to their communities. Flyers and other information, education and communication (IEC) activities were designed together with community members in general (some of whom may have also been peer educators). Peer educators met participants where they were available and came up with targeted approaches based on the different communities. They played an important role in addressing concerns and tailoring messages. Early adopters, known as “guides” or “PrEP ambassadors” also shared their experiences taking PrEP. Peer educators played a key role in retaining participants in the study and were responsible for checking in with participants who missed appointments to understand the reasons why and address them jointly with the health care workers at participating facilities. Regular meetings were held between peers and organizational staff to discuss this work. Support group meetings were held monthly and provided opportunities for participants to share challenges with PrEP and discuss potential solutions. Attendance and structure of support groups varied by site, as some were extensions of pre-existing support groups for HIV negative individuals enrolled in prevention programs. Challenges addressed included adherence modelling by good adherers, side effects and social dynamics such as dealing with stigma, and the effect of PrEP use on personal relationships.

At one point during implementation, young men in one of the urban sites stormed LVCT offices and demanded a stop of the project alluding that it was providing their YW a drug that would make them infertile. LVCT halted the project for a few days and used this time to engage the wider community, including young men who were not a target population. The teams worked with the site CAC, CBOs, local village administrative chiefs, families, male opinion leaders, and partners of the participants, to increase awareness about PrEP and educate the wider community to support enrollment and retention. The project continued to keep community members aware of the progress and undertook regular community awareness activities such as utilizing the local area Chief’s community meetings, participating in talk shows at the local radio station, and utilizing a community-level HIV prevention champions to talk about PrEP and the project.

The project was successful in identifying individuals at risk of HIV infection for PrEP initiation and demonstrating the effectiveness of PrEP delivery in real world settings. The lessons learned during project implementation informed the national PrEP policy and PrEP scale up in Kenya. The project provided support to the national PrEP rollout, including informing national communication strategies, adapting the project clinical evaluation and training curriculum for providers and peer educators into national tools, informing community stakeholder mapping and coordination, implementation planning, and PrEP advocacy. As PrEP is now available in public and community-based facilities in Kenya, LVCT Health and other implementing partners work with the Government to engage communities in demand generation, awareness creation, and adherence support during PrEP scale up. Community ambassadors continue to play a critical role in scaling up PrEP in Kenya.

## The Treatment And Prevention for Female Sex Workers (TAPS) Demonstration Project: South Africa

The TAPS Demonstration Project was conducted by Wits RHI between March 2015 and July 2017, in Johannesburg and Pretoria, South Africa ^30^. The project was embedded in Wits RHI’s well-established Sex Worker Project (SWP). FSWs accessing HIV, STI, and reproductive health services, as well as FSWs from the surrounding communities were invited to participate in the study. Post-screening, eligible FSWs were either offered ART or PrEP along with the range of Wits RHI clinic services through the SWP. The main aim of the project was to assess the feasibility of providing PrEP as part of combination prevention packages and early ART as part of an HIV intervention to FSWs in urban settings in South Africa. Overall, 351 women were screened and 219 were enrolled for the PrEP arm of the study.

Meetings with community organizations, FSWs, and other stakeholders, such as sex worker groups SWEAT and Sisonke, Sonke Gender Justice, the Women’s Legal Centre (WLC), Treatment Action Campaign (TAC), and police representatives, started in the summer of 2013 ^31^. Four focus group discussions were help in each site to assess the community acceptability of PrEP ^32^. The discussions showed high acceptability and anticipation of PrEP. This formative research developed the basis for the intervention. Community engagement was conceived from the notion that PrEP was a fairly new intervention in South Africa, both the community and FSWs had no knowledge of PrEP, making it essential to create awareness of both PrEP and the study among the community and potential study participants (FSWs). Community engagement provided a platform for the project to understand the needs of the community and for the community to give input on the direction of the project. A ten-person CAB, comprised of representatives from the organizations and two-to-three FSWs, was formed in 2014. The CAB meetings were used as a platform for identifying any training needs of CAB members, advising research staff on sex worker community norms and expectations, and creating a supportive environment for the project by raising awareness and dispelling myths/rumors about PrEP. CAB members also facilitated recruitment by identifying contacts. The CAB chairperson was from a sex worker organization and meetings were convened monthly.

The TAPS study collaborated with the existing peer educators from the SWP who were instrumental in delivering education about PrEP by incorporating PrEP messages into their existing peer outreach activities, getting buy-in by the community, and identifying high-risk FSWs from their network. The project worked with 12 peer educators from the SWP who were selected based on their social networks and their experience in conducting outreach. Peer educators were responsible for follow up. Little was known about PrEP at the start of the study, so a lot of initial work focused on demystifying PrEP, educating the community, and getting buy-in. Cards with the project details were used for recruitment. Recruitment was done during day and night, in streets, brothels, hotels, and clinics—where peers would often accompany participants to their screening. During outreach, peer educators conducted education sessions and handed information materials to women including condom bags and discussed various issues that impacted them, such as police harassment, client abuse, violence, and STIs. Research staff attended these sessions to provide information about PrEP and respond to any questions raised. A mobile clinic service was established to access and create demand for sex workers who were based farther away from the clinics. The mobile clinic was leveraged from the SWP clinic to recruit FSWs who were not able to access the clinic either because of distance or due to the clinic operating hours. These processes facilitated recruitment.

The peer educators played a key role in supporting participants who missed follow up appointments, by addressing concerns such as side effects and challenges with partners. Peers were best able to draw on their own experiences when addressing concerns. During the study, several challenges emerged including xenophobic riots and police raids where many participants worked and lived. Engagement with sex worker advocacy groups supported the sex workers affected by these issues. The sex worker advocacy groups were not always successful in supporting the sex workers, particularly in areas where there were no firm relationships with the police. Sensitizing the police and creating awareness of FSW needs amongst the police is an ongoing intervention in creating an enabling environment for FSWs to access healthcare and social and structural services.

The TAPS study was the first project to offer PrEP to FSWs in South Africa. Following the project, the participants were rolled over to the SWP, where they are currently still receiving services. Due to the involvement of the CAB and longer-term community engagement, the community demanded that PrEP should be available more widely (beyond TAPS). An important outcome of the project was that the national program began initiating PrEP for sex workers in 11 clinics across South Africa, even before the end of the study. This was largely possible due to the constant feedback and reporting to the National Department of Health from the research team regarding their learning and experiences on a rolling basis. The CAB members, including some peer educators, were involved in the dissemination of the TAPS results. Results and lessons learned from the TAPS project were used to develop the first set of national guidelines to include PrEP, which were initially focused on the rollout to sex workers ^24^.

## India: The Closing a Critical Prevention Gap project

The Closing a Critical Prevention Gap project took place at two sites in India to evaluate PrEP delivery strategies, monitor adherence to and discontinuation of PrEP, and evaluate unintended consequences of the use of PrEP in these communities. The University of Manitoba, as the technical partner, supported two well-established community-based organizations that implemented demonstration projects in two distinctive settings. As sex work organizations led the implementation, peer leaders and educators took on central roles in all aspects of the project from design to implementation.

## Durbar Mahila Samanwaya Committee (DMSC) case study: Kolkata, India

Starting in 1992, the Sonagachi Project in Kolkata began providing HIV prevention services for FSWs. It soon grew into the Durbar Mahila Samanwaya Committee (DMSC), a community-owned sex work project of more than 60,000 FSWs. The PrEP demonstration project began by raising awareness on PrEP steered by the members of the DMSC along with the peer educators of the ongoing HIV intervention program. A feasibility study was conducted to identify possible enablers and barriers in taking PrEP. Screening for the demonstration project started in January 2016. A total of 843 FSWs were screened out of which 678 enrolled. Peer educators were trained to discuss condoms and PrEP as “double protection”. Fifteen peer educators were trained and each trained peer was responsible for following fifty sex workers. Based on the feasibility study, a potential participant list was created of women interested in PrEP and divided among the peer educators.

Although women were interested in taking PrEP, during recruitment it was found that some madams (brokers) would not let them go through the screening process as it would waste a day of work. This reinforced the importance of working with other stakeholders, including pimps and boyfriends, in addition to madams. Peer educators provided stakeholders with information on PrEP and how it prevents HIV and therefore helps maintain health, which then helps ensure the ability to keep working and earning an income. To create interest, the cultural team of DMSC performed street dramas in Sonagachi, to promote the effectiveness of PrEP as a combination HIV prevention tool.

Peer educators played a vital role in walking participants through the screening and follow up. They would initially visit participants every day for the first 7 days to ensure daily intake and monitor side effects. Counselors and doctors also made home visits. Once adjusted to PrEP, peer educators started providing medicine weekly, fortnightly, and then monthly, following the first month clinical follow up. Telephone calls were also used to remind women of their follow up visits.

One of the major challenges identified during the project was the frequent travel of sex workers to visit with their children, as well as frequent movement from Sonagachi to other red light districts. During travel, the women would often not carry PrEP with them for fear of being identified as sex workers or being misconstrued as living with HIV. Another challenge was faced from partners, boyfriends, or regular clients who also wanted access to PrEP, with some taking PrEP from the sex workers. While challenges with frequent travel was addressed by asking about upcoming travel plans and providing extra pills if travel was planned before the next refill date, it also may have proven useful to form a “Campaign Team” comprised of different stakeholders (madams, landladies, and fixed clients) to address some of these challenges more effectively.

## Ashodaya Samithi case study: Mysore, India

Ashodaya Samithi, a female, male, and transgender sex work collective, implemented the PrEP project in Mysore-Mandya. The project began with awareness sessions and a feasibility study, between December 2014 and May 2015 ^33^. The demonstration project ran from March 2016 until February 2018. In total, 647 participants were enrolled after screening 707 FSWs.

The sex work community played key roles in all phases of the design and implementation of the study. Numerous awareness sessions and small group discussions were held in the field to answer all questions about PrEP and understand community concerns. These sessions were led by Ashodaya community leaders. Discussions surrounded the need for PrEP, condom use, side effects, travel, and alcohol use. Community members expressed a great need for PrEP, especially in instances where they were unable to use condoms.

The sites were divided into 10 clusters with 20 community mobilizers playing a key role in the recruitment and follow up of the participants. Community mobilizers were existing Ashodaya outreach workers who received additional training on PrEP and were purposively chosen to represent and support different networks. Following recruitment, mobilizers accompanied potential participants to screening visits. Negotiations took place with the lab for same-day test results as to not lose potential participants to follow up. Once enrolled, community mobilizers were responsible for following up with participants from their network to ensure delivery of PrEP fortnightly, monitor adherence and routine quarterly clinic visits, and address any crises that may have arose. Daily check-ins provided opportunities to offer support for other health and social issues beyond PrEP to both participants taking PrEP and their wider networks.

Sex work community leaders and mobilizers, who are longstanding members of the organization and were involved in the discussions surrounding initiating and planning the demonstration project, volunteered to be the first participants enrolled in the study, which built trust in the project. Leaders then went on to recruit and mobilize participants through their peer networks by acting as “role models” and sharing their personal testimonials taking PrEP. Sex work leaders living with HIV further supported the usefulness of PrEP and were involved in mobilization to ensure that the study did not create social divisions in the community between those living with HIV and those who were HIV negative. Rumors about PrEP and side effects were addressed by peers at the ground level. Attempts were made to address all concerns as they arose. Sex work leaders and the technical support team conducted sensitization sessions with the police to create an enabling environment for the project and help prevent disruptions in adherence due to raids and arrests. This helped in building community trust and buy-in. Similar sensitization sessions were held with boyfriends, clients, family, and brokers as they too played key roles in ensuring retention.

The demonstration projects in India were well received by participants and demand for ongoing PrEP post-demonstration project continues. A number of the participants in both sites were able to continue PrEP after their exit from the study, due to a surplus in tablets, however this supply has since ended. Press and electronic media coverage is being used by the organizations to disseminate findings and to help advocate for PrEP to be included as part of the national HIV prevention package in India.

## Discussions and lessons learned

These case studies highlight the important role that community engagement played in the PrEP demonstration projects. As many countries are interested in initiating PrEP or scaling up PrEP access ^24^, it is important to understand the best ways to implement PrEP as part of combination HIV prevention. The case studies document various aspects of PrEP implementation and highlight the programmatic approaches undertaken to deliver PrEP to two target populations: serodiscordant couples and key population that includes FSWs, MSM, and YW. Although there were context specific considerations in the design of the interventions, there are some commonalities between projects that are important lessons for PrEP roll out.

In the demonstration projects among serodiscordant couples, the community was broadly defined. It extended beyond the primary stakeholders, the HIV serodiscordant couples themselves, to include local leaders, religious leaders, and local community organizations. Key to initiating PrEP among serodiscordant couples is the promotion of couple’s testing. Mass media communication strategies that included the use of television, radio, and street theatre, played an important role in promoting couple’s counseling and testing, and included targeted messages to men, who often show more reluctance to testing ^34– 36^. Serodiscordant couples groups should play a critical role in providing this support, along with dispelling myths/misconceptions about PrEP, and offering adherence support. Simultaneously, capacity building for health care workers when planning PrEP access is also important ^37^. Thus, it is key to develop multi-pronged strategies of community engagement, capacity building, and mass media promotion to generate demand when implementing PrEP among serodiscordant couples.

The demonstration projects documented the critical role of community, defined here mainly as FSWs, MSM, and YW who are at risk of getting HIV. The local context was a determining factor for the level of community involvement in planning and implementing the community engagement program for these populations. Despite variations in the design and implementation of the community engagement program between sites, several commonalities were observed. Each of the locations made sure that community leaders and peer educators played a role in developing education materials and tools that promoted PrEP awareness and demand. Also, as observed in the projects conducted with HIV serodiscordant couples, projects among sex workers also reached wider community and stakeholders who could influence decisions around PrEP use and adherence, such as madams, boyfriends, clients, and the police. This highlights the need to engage communities beyond just the intended users of PrEP in order to create an enabling environment that supports PrEP access and use.

Taking lessons from the proven strategies of peer-led outreach in HIV prevention and treatment programs ^38– 41^, these studies highlight how communities play strategic roles ranging from demand generation, to PrEP distribution among their social networks, and ensuring follow up ( Box 1). Community leaders or peer educators were often the “early adopters,” who became “role models” in ensuring adherence. Some of these case studies highlight that the demonstration projects were steered and owned by the community through their community-based organizations. This points to the importance of providing sufficient resources to support community engagement in implementing PrEP projects.

Box 1. **Elements of Successful Community Engagement**

- Community advisory committees to oversee the design and implementation of the study
- Community involvement in awareness raising, information sessions, and demand generation
- Community leaders as early adopters and role models
- Peer educators involved in recruitment, screening, follow up, adherence support, and addressing challenges
- Community-led dissemination events

While the case studies presented here are limited in scope, they provide important insight into the ways that communities are engaged in PrEP demonstration projects globally. As each site developed contextually-specific approaches to rolling out their demonstration projects, comparison of the case studies could not be done. Yet, context-specific designs were important for the community engagement programs to be successful. Therefore, these studies provide important insights on ways to engage communities in biomedically-focused research and programs. More detailed findings from each site will be published separately.

## Conclusion

The WHO underpins the importance of PrEP as an HIV prevention option ^3^ and the importance of community engagement in implementing PrEP ^42^. Several countries, such as Kenya, South Africa, and Nigeria, have initiated PrEP scale-up post-demonstration projects. As further countries move forward with PrEP implementation, it is critical to understand how best to scale up PrEP in diverse settings. Community engagement has emerged as a critical factor for education, demand generation, dispelling rumors, and supporting adherence and follow up in the PrEP demonstration project case studies. The increasing global interest in PrEP necessitates the importance of understanding the best ways to implement PrEP in different settings as part of combination HIV prevention. PrEP as a biomedical solution has to have a huge buy-in by the community – from its leadership to its larger networks who go on to become the users of this important prevention tool.

## Data availability

No data are associated with this article.
