# Supplementary material for: Community Inclusion in PrEP Demonstration Projects: Lessons for Scaling Up
Source: Gates Open Res. 2019 Dec 17;3:1504. Originally published 2019 Jul 5. [Version 2] doi: 10.12688/gatesopenres.13042.2 (PMC6943951; doi:10.12688/gatesopenres.13042.2)
Supplement: Peer review history

# Peer review: Reviewer response for version 2

**DOI:** 10.21956/gatesopenres.14248.r28379

**Published:** 2019-12-30

**Authors:** Paul Ndebele

**Affiliations:** 1 Medical Research Council of Zimbabwe, Harare, Zimbabwe; 2 The George Washington University, Washington, DC, USA

The authors have already made significant improvements to the manuscript based on the comments from the other two reviewers and they have in some ways addressed my main concerns. I recommend that the manuscript be accepted.

I confirm that I have read this submission and believe that I have an appropriate level of expertise to confirm that it is of an acceptable scientific standard.

# Peer review: Reviewer response for version 1

**DOI:** 10.21956/gatesopenres.14158.r27496

**Published:** 2019-08-13

**Authors:** Kathleen M. MacQueen

**Affiliations:** 1 Global Health Research, FHI 360, Durham, NC, USA; 2 University of North Carolina Center for AIDS Research, Chapel Hill, NC, USA; 3 Social Medicine Department, School of Medicine, University of North Carolina at Chapel Hill, Chapel Hill, NC, USA

Summary:

- The open letter is a valuable description of the community engagement practices implemented in seven BMGF-funded PrEP demonstration projects in various sites in Kenya, Uganda, Nigeria, Benin, Senegal, South Africa, and India. The projects focused primarily on serodiscordant couples and female sex workers and were variously implemented by in-country and expat research centres, as well as community-based and advocacy organizations. Direct governmental participation in the projects is unclear and it would be helpful to have that clarified.
- The projects varied with regard to the community engagement strategies used. Using data from an open-ended questionnaire completed by members of each project and follow-up phone interviews with some projects, case studies were developed describing the role of community at various stages of the projects. With input from the projects, the case studies were then assessed for commonalities and lessons learned.
- The rationale for the open letter is clearly articulated in the Background section. The authors cite the controversial history of early PrEP trials that led to more proactive community engagement. They note the importance of sustaining community participation in the context of PrEP demonstration projects, especially given the diversity of settings and at-risk populations. The letter seeks to add to the documentation of practice and lessons learned across the diverse settings, providing a range of views and experiences on the topic.

 

Major points to address:

- To more adequately represent the diversity of opinion on community engagement (CE) in the projects, it would be helpful if the authors included a statement describing how each project conceptualized the role of CE in their project and how success was defined by each project. This information is woven into the case studies (for the most part) but the reader would benefit from articulation of role/desired outcomes in the opening paragraph for each case study.
- The richness of the case studies is quite variable, and it is difficult to determine if this is due to the complexity or comprehensiveness of the CE work across projects/sites or due to limitations in the data collected for each case study. Clarification on this is needed.
- The descriptions of post-project outcomes related to sustainability of PrEP access, dissemination, PrEP advocacy, and on-going work related to community engagement in these processes is very helpful. Explicit commentary on barriers and challenges would also be helpful, especially if any of the projects have reflected on what they would do differently based on their experience. Some of this alluded to but not explicitly addressed, making it difficult for the reader to parse across the case studies.
- P. 3, under Findings: The opening paragraph lists populations targeted but omits MSM and young women, who are described as participants in some case studies.
- P. 4, Table 1: The Implementer column is ambiguous as to whether the organizations listed were responsible for the PrEP demonstration project, the community engagement work, or both. Clearer understanding of the relative independence of the CE work for each project would help the reader and potentially add insight to the lessons learned.
- P. 4, Partners Project: From the description it appears that the CHWs had built considerable trust with the community prior to their engagement with the project. The project was then able to leverage this existing trust — an important nuance. A key question then becomes, how did implementers build trust with the CHWs? CHWs risked undermining the community trust they previously established, if they did not negotiate trust-building with the implementers. This is important context to understand for lessons learned.
- P. 6, second full paragraph: The last sentence states that CWs in Benin “simultaneously addressed concerns in the community” while working one-on-one with project participants. What strategies did the CWs use to address community concerns? What types of concerns emerged? How were they addressed?
- Pp. 6-7, IPCP project: It is unclear whether or how the project CAB and site level community advisory committees interacted or had overlapping membership; this should be clarified. Also, to what extent were these two types of advisory groups engaged in addressing the LVCT crisis when the young men stormed the offices? Was representation at the site level expanded to include young men? Or was expanded stakeholder engagement distinct from the established advisory mechanisms?
- P. 7, TAPS project: It is unclear whether the mobile clinic service for sex workers was established specifically to facilitate project recruitment, or to address unmet needs for sex workers generally. I.e., was the mobile clinic exploiting unmet needs for recruitment purposes or was the project leveraged to address those needs with an eye toward sustainability beyond the project?
- P. 8, Ashodaya Samithi project: The authors state that sex work community leaders were the first participants who then “went on to recruit and mobilize participants through their peer networks.” To what extent was this recruitment/mobilization role fostered by the project staff? Were the leaders an integral part of the sex work collective implementing the project? If not, were they paid for taking on the recruitment/mobilization role? How was their role related to the community mobilizers, who are also described as having major roles in recruitment and follow-up?
- Discussion section: It would be helpful to include an explicit discussion of gender power dynamics and how these were handled in the various projects. Some sites broadened outreach to stakeholders with power/authority over young women (YW) and FSWs in particular, to gain the support of such stakeholders for participation of YW/FSW in the PrEP programmes. Gender integration is often conceptualized as a continuum from exploitation to accommodation to transformation of inequalities/inequities. To what extent were projects consciously addressing this continuum in their choice of strategies? What lessons emerged with regard to gender integration in the various PrEP programmes?

I confirm that I have read this submission and believe that I have an appropriate level of expertise to confirm that it is of an acceptable scientific standard, however I have significant reservations, as outlined above.

# Response

**Published:** 2019-12-04

**Authors:** Lisa Lazarus

- The open letter is a valuable description of the community engagement practices implemented in seven BMGF-funded PrEP demonstration projects in various sites in Kenya, Uganda, Nigeria, Benin, Senegal, South Africa, and India. The projects focused primarily on serodiscordant couples and female sex workers and were variously implemented by in-country and expat research centres, as well as community-based and advocacy organizations. Direct governmental participation in the projects is unclear and it would be helpful to have that clarified.
- The projects varied with regard to the community engagement strategies used. Using data from an open-ended questionnaire completed by members of each project and follow-up phone interviews with some projects, case studies were developed describing the role of community at various stages of the projects. With input from the projects, the case studies were then assessed for commonalities and lessons learned.
- The rationale for the open letter is clearly articulated in the Background section. The authors cite the controversial history of early PrEP trials that led to more proactive community engagement. They note the importance of sustaining community participation in the context of PrEP demonstration projects, especially given the diversity of settings and at-risk populations. The letter seeks to add to the documentation of practice and lessons learned across the diverse settings, providing a range of views and experiences on the topic.

*- We thank the reviewer for their feedback on our open letter and have provided detailed responses to the specific queries below.*

Major points to address:

- To more adequately represent the diversity of opinion on community engagement (CE) in the projects, it would be helpful if the authors included a statement describing how each project conceptualized the role of CE in their project and how success was defined by each project. This information is woven into the case studies (for the most part) but the reader would benefit from articulation of role/desired outcomes in the opening paragraph for each case study.

*-Thank you for raising this important point. We have included statements on how each project conceptualized community engagement at the start of each case study, following the overall objective, and also worked to describe project success towards the end of each case study.*

 

- The richness of the case studies is quite variable, and it is difficult to determine if this is due to the complexity or comprehensiveness of the CE work across projects/sites or due to limitations in the data collected for each case study. Clarification on this is needed.

*-Thank you for this query. The variability is due to the different levels of engagement, based on the existing roles of communities in each environment. We have attempted to address this in the Discussion with the following statement: “While the case studies presented here are limited in scope, they provide important insight into the ways that communities are engaged in PrEP demonstration projects globally. As each site developed contextually-specific approaches to rolling out their demonstration projects, comparison of the case studies could not be done. Yet, context-specific designs were important for the community engagement programs to be successful. Therefore, these studies provide important insights on ways to engage communities in biomedically-focused research and programs.”*

- The descriptions of post-project outcomes related to sustainability of PrEP access, dissemination, PrEP advocacy, and on-going work related to community engagement in these processes is very helpful. Explicit commentary on barriers and challenges would also be helpful, especially if any of the projects have reflected on what they would do differently based on their experience. Some of this alluded to but not explicitly addressed, making it difficult for the reader to parse across the case studies.

*-Thank you for raising this important point. We have added details about challenges faced and whether there are some steps that projects would have done differently to the case studies. *

- P. 3, under Findings: The opening paragraph lists populations targeted but omits MSM and young women, who are described as participants in some case studies.

*-Thank you for catching this. It was been corrected.*

 

- P. 4, Table 1: The Implementer column is ambiguous as to whether the organizations listed were responsible for the PrEP demonstration project, the community engagement work, or both. Clearer understanding of the relative independence of the CE work for each project would help the reader and potentially add insight to the lessons learned.

*- All implementers were involved in leading the project. Where appropriate, community organizations involved in all aspects of study implementation have also been included in the Table. Details of community partners involved in some areas of community engagement are included in the case studies.*

- P. 4, Partners Project: From the description it appears that the CHWs had built considerable trust with the community prior to their engagement with the project. The project was then able to leverage this existing trust — an important nuance. A key question then becomes, how did implementers build trust with the CHWs? CHWs risked undermining the community trust they previously established, if they did not negotiate trust-building with the implementers. This is important context to understand for lessons learned.

*-We have clarified that the Partners PrEP demonstration project builds on the previous PrEP trial. CHWs had been involved in the previous trial, therefore the demonstration project builds from this previous work and previously developed relationships.*

- P. 6, second full paragraph: The last sentence states that CWs in Benin “simultaneously addressed concerns in the community” while working one-on-one with project participants. What strategies did the CWs use to address community concerns? What types of concerns emerged? How were they addressed?

*- Thank you for this question. We have revised the Benin case study to provide more details. Specifically, we have clarified that catchment areas were divided in a way that allowed for CWs, who were from NGOS working with sex workers, to frequently meet and support FSWs in the field. CWs worked to promote adherence, addressed concerns about side effects, and located women lost to follow up during their field visits.*

- Pp. 6-7, IPCP project: It is unclear whether or how the project CAB and site level community advisory committees interacted or had overlapping membership; this should be clarified. Also, to what extent were these two types of advisory groups engaged in addressing the LVCT crisis when the young men stormed the offices? Was representation at the site level expanded to include young men? Or was expanded stakeholder engagement distinct from the established advisory mechanisms?

*-We have clarified the different memberships and roles of the national-level CAB, as compared to the site and population specific community advisory committees in the IPCP case study. We have also specified that both the CAB and CAC were involved in addressing the crisis, along with engaging other important stakeholders.*

 

- P. 7, TAPS project: It is unclear whether the mobile clinic service for sex workers was established specifically to facilitate project recruitment, or to address unmet needs for sex workers generally. I.e., was the mobile clinic exploiting unmet needs for recruitment purposes or was the project leveraged to address those needs with an eye toward sustainability beyond the project?

*-Thank you for this query. We have clarified the above with the following statement: “A mobile clinic service was established to access and create demand for sex workers who were based farther away from the clinics. The mobile clinic was leveraged from the SWP clinic to recruit FSWs who were not able to access the clinic either because of distance or due to the clinic operating hours. These processes facilitated recruitment.”*

- P. 8, Ashodaya Samithi project: The authors state that sex work community leaders were the first participants who then “went on to recruit and mobilize participants through their peer networks.” To what extent was this recruitment/mobilization role fostered by the project staff? Were the leaders an integral part of the sex work collective implementing the project? If not, were they paid for taking on the recruitment/mobilization role? How was their role related to the community mobilizers, who are also described as having major roles in recruitment and follow-up?

*-Thank you for this question. As Ashodaya is a sex work-led organizations, community leaders took on central roles in all aspects of the project from design to implementation. We have clarified that: “Sex work community leaders and community mobilizers, who are longstanding members of the organization and were involved in the discussions surrounding initiating and planning the demonstration project, volunteered to be the first participants enrolled in the study, which built trust in the project.”*

 

- Discussion section: It would be helpful to include an explicit discussion of gender power dynamics and how these were handled in the various projects. Some sites broadened outreach to stakeholders with power/authority over young women (YW) and FSWs in particular, to gain the support of such stakeholders for participation of YW/FSW in the PrEP programmes. Gender integration is often conceptualized as a continuum from exploitation to accommodation to transformation of inequalities/inequities. To what extent were projects consciously addressing this continuum in their choice of strategies? What lessons emerged with regard to gender integration in the various PrEP programmes?

*-While we agree that an analysis of gender power dynamics would make an important contribution to this work, it is beyond the scope of this open letter. The independent project sites will explore this in their own site-specific papers.*

# Peer review: Reviewer response for version 1

**DOI:** 10.21956/gatesopenres.14158.r27498

**Published:** 2019-07-30

**Authors:** Darrell H.S. Tan, Robinson Truong

**Affiliations:** 1 Division of Infectious Diseases, St. Michael's Hospital, Toronto, ON, Canada; 2 St. Michael's Hospital, Toronto, Ontario, Canada

This open letter is a narrative review describing community engagement activities undertaken within seven PrEP demonstration projects funded by the Gates foundation. The authors rightly emphasize the importance of inclusive community engagement in such studies by citing the lack of such work in the early tenofovir trials. Overall the article provides a concise summary and includes descriptions that may be of interest to researchers planning future HIV prevention or global health intervention studies. A few suggestions follow.

 Major comments:

1. It would be helpful for the abstract to mention some of the key actual lessons learned, rather than only stating that lessons learned are included in the body of the article.
2. It would be helpful if each of the case studies were structured in a similar way and contained the same key elements. At present, most open with a paragraph outlining who the study implementers were, and then describe who the key actors were for the community engagement, specific activities undertaken by those community-based actors, challenges encountered, and commentary about the current status of PrEP funding in the local setting. In particular, only a minority of the case studies clearly describe challenges faced by the study teams in working with community, which may be of particular interest to those hoping to learn from these experiences. Also, statements about the availability and cost of PrEP in some of the examples could be clearer (e.g. India, Partners does not comment on Uganda). Finally, it would be appropriate to add a clear brief statement about the overarching goal or primary objective for each project; this was often but not always done.
3. To make the case studies as useful as possible for readers interested in implementing similar community engagement work, it would be helpful for the case studies to be as specific as possible, perhaps including operational details such as exactly how the key actors (CAB members, community workers, peer educators etc.) were selected, how many were engaged and why, and specific activities undertaken by these individuals. In some cases, relevant details are lacking, making the information potentially less useful to readers. For instance, the Partners PrEP description contains a series of somewhat vague statements about a variety of community actors doing various general types of activities, without really specifying who was responsible for taking on which specific actions; e.g. there is mention of lead members of community organizations, CAGs, CHWs, and serodiscordant couple groups, but the rationale for and differences between these agents was not clear, and there is separate mention in different places of having “worked with stakeholders to dispel myths, rumours etc”, being “agents who interacted with the local communities”, “regular engagements with various stakeholders” etc.  Similarly, in the Nigerian case study, many activities are described in the passive voice, making it again less clear which actors undertook which activities (“letters were sent…”, “monthly meetings were held…” etc); presumably many of these were activities organized by the investigators to support the community partners but this could be clearer.
4. The authors could consider including a box or Table that lists common key elements of successful community engagement in these diverse projects, with the goal of helping readers see at a glance what specific advantages there may be to such work, and how it might be approached.   
                

Minor comments:

1. The introductory paragraph could specify the particular tenofovir prodrug as TDF (versus TAF).
2. In the background, it may be appropriate to briefly mention the ethical violations that are alluded to (and cited) in the second paragraph.
3. At the end of the background, it might be helpful to state that the purpose of the paper is not only to articulate the community engagement practices implemented in the projects but also to explicitly inform future work with appropriate community engagement/advise other investigators on how to do such work.
4. The Partners PrEP description contains a somewhat confusing statement about how community entry was initiated by seeking government approval; what exactly was meant by this?  
5. Later in the same paragraph the authors say that “site projects instituted CAGs”; is this a typo that should have read “project sites”?
6. When describing lessons learned, the Discussion mentions that special attention should be paid to handling “crises” in serodiscordant couples, but this concept was not really explained in the case studies themselves (except when mentioning that some couples faced “challenges” in Partners PrEP).

We confirm that we have read this submission and believe that we have an appropriate level of expertise to confirm that it is of an acceptable scientific standard, however we have significant reservations, as outlined above.

# Response

**Published:** 2019-12-04

**Authors:** Lisa Lazarus

This open letter is a narrative review describing community engagement activities undertaken within seven PrEP demonstration projects funded by the Gates foundation. The authors rightly emphasize the importance of inclusive community engagement in such studies by citing the lack of such work in the early tenofovir trials. Overall the article provides a concise summary and includes descriptions that may be of interest to researchers planning future HIV prevention or global health intervention studies. A few suggestions follow.

*We thank the reviewer for their feedback on our open letter and have provided detail responses to the specific queries below.*

Major comments:

1. It would be helpful for the abstract to mention some of the key actual lessons learned, rather than only stating that lessons learned are included in the body of the article.

*-We have added lessons learned to the abstract.*

2. It would be helpful if each of the case studies were structured in a similar way and contained the same key elements. At present, most open with a paragraph outlining who the study implementers were, and then describe who the key actors were for the community engagement, specific activities undertaken by those community-based actors, challenges encountered, and commentary about the current status of PrEP funding in the local setting. In particular, only a minority of the case studies clearly describe challenges faced by the study teams in working with community, which may be of particular interest to those hoping to learn from these experiences. Also, statements about the availability and cost of PrEP in some of the examples could be clearer (e.g. India, Partners does not comment on Uganda). Finally, it would be appropriate to add a clear brief statement about the overarching goal or primary objective for each project; this was often but not always done.

*-Thank you for this important comment. We have worked to revise the case studies to ensure that they all present the overall purpose of the project, key actors involved, activities undertaken for community engagement, challenges faced, and project outcomes as they relate to the current status of PrEP in each site. In particular, we have included challenges faced and addressed in the different projects. We have also clarified current PrEP status in India and Uganda.*

3. To make the case studies as useful as possible for readers interested in implementing similar community engagement work, it would be helpful for the case studies to be as specific as possible, perhaps including operational details such as exactly how the key actors (CAB members, community workers, peer educators etc.) were selected, how many were engaged and why, and specific activities undertaken by these individuals. In some cases, relevant details are lacking, making the information potentially less useful to readers. For instance, the Partners PrEP description contains a series of somewhat vague statements about a variety of community actors doing various general types of activities, without really specifying who was responsible for taking on which specific actions; e.g. there is mention of lead members of community organizations, CAGs, CHWs, and serodiscordant couple groups, but the rationale for and differences between these agents was not clear, and there is separate mention in different places of having “worked with stakeholders to dispel myths, rumours etc”, being “agents who interacted with the local communities”, “regular engagements with various stakeholders” etc.  Similarly, in the Nigerian case study, many activities are described in the passive voice, making it again less clear which actors undertook which activities (“letters were sent…”, “monthly meetings were held…” etc); presumably many of these were activities organized by the investigators to support the community partners but this could be clearer.

*-We have added more details to each of the case studies, including how key actors were selected for different community engagement strategies and the number of people engaged in different activities. This includes revisions to both the Partners and Nigerian case studies, in order to provide further clarity to the different activities.*

4. The authors could consider including a box or Table that lists common key elements of successful community engagement in these diverse projects, with the goal of helping readers see at a glance what specific advantages there may be to such work, and how it might be approached.   

*-Thank you for this suggestion. We have added Box 1 in the Discussion section that highlights the common key elements of community engagement.   *           

Minor comments:

1. The introductory paragraph could specify the particular tenofovir prodrug as TDF (versus TAF).

*-This has been added.*

2. In the background, it may be appropriate to briefly mention the ethical violations that are alluded to (and cited) in the second paragraph.

*-Details on the ethical violations of early tenofovir trials have been added.*

3. At the end of the background, it might be helpful to state that the purpose of the paper is not only to articulate the community engagement practices implemented in the projects but also to explicitly inform future work with appropriate community engagement/advise other investigators on how to do such work.

*-A statement on the purpose of the paper has been added to the end of the Background section.*

4. The Partners PrEP description contains a somewhat confusing statement about how community entry was initiated by seeking government approval; what exactly was meant by this?  

*-Thank you for pointing this out. The sentence has been edited to read: “Approval for the project was initially sought from national and local government authorities in the Ministry of Health and Public Works, which was followed by meetings with lead members of community organizations (non-governmental, community-based, and faith-based), HIV serodiscordant couples support groups, and national and local health care authorities.” Further details on the formation of the community advisory groups has also been added to the case study.*

5. Later in the same paragraph the authors say that “site projects instituted CAGs”; is this a typo that should have read “project sites”?

*-Thank you for catching this. This has been corrected.*

6. When describing lessons learned, the Discussion mentions that special attention should be paid to handling “crises” in serodiscordant couples, but this concept was not really explained in the case studies themselves (except when mentioning that some couples faced “challenges” in Partners PrEP).

*- We have removed this sentence as it has not been explored in the studies presented.*

# Peer review: Reviewer response for version 1

**DOI:** 10.21956/gatesopenres.14158.r27497

**Published:** 2019-07-26

**Authors:** Paul Ndebele

**Affiliations:** 1 Medical Research Council of Zimbabwe, Harare, Zimbabwe; 2 The George Washington University, Washington, DC, USA

- The authors state that their intention is to describe CE strategies and lessons learned. Much of the paper reads like a progress report on recruitment and retention strategies. CE is more than that. The reader expects them to focus on strategies used, why they were selected, how useful the strategies were, and what the challenges associated with those selected strategies were. They have not even succeeded in fully articulating the various roles of the communities in the various projects. What strategies did they employ to influence acceptance and community involvement? The sections on South Africa, Kenya/Uganda and Nigeria describe some specifics on strategies. 
- The discussion section also focuses very much on recruitment and retention. 
- The conclusion section focuses more on PrEP roll-out than on CE. The topic could be edited to focus on community engagement strategies in PrEP demonstration projects. 
- The sub-heading on "Assessment of the research projects” is misleading as the section describes documentation of CE strategies/practices.
- In the Table no sites are stated for Nigeria and some abbreviations are used for some implementers. Based on what happened to previous HIV prevention trials, CE should therefore focus on acceptance and keeping communities updated about trials and addressing concerns and misconceptions/rumours.

I confirm that I have read this submission and believe that I have an appropriate level of expertise to state that I do not consider it to be of an acceptable scientific standard, for reasons outlined above.
